# Size of carotid artery intraplaque hemorrhage and acute ischemic stroke: a cardiovascular magnetic resonance Chinese atherosclerosis risk evaluation study

**DOI:** 10.1186/s12968-019-0548-1

**Published:** 2019-07-01

**Authors:** Yang Liu, Maoxue Wang, Bing Zhang, Wei Wang, Yun Xu, Yongjun Han, Chun Yuan, Xihai Zhao

**Affiliations:** 1 Department of Radiology, The Affiliated Hospital of Yangzhou University, Yangzhou, China; 2 Department of Biomedical Engineering, Center for Biomedical Imaging Research, Tsinghua University School of Medicine, Beijing, China; 3 Department of Radiology, The Affiliated Drum Tower Hospital of Nanjing University Medical School, Nanjing, China; 4 Department of Medical Imaging, Taikang Xianlin Drum Tower Hospital, Nanjing, China; 5 Department of Neurology, The Affiliated Drum Tower Hospital of Nanjing University Medical School, Nanjing, China; 6 Center for Brain Disorders Research, Capital Medical University and Beijing Institute for Brain Disorders, Beijing, China; 7 Department of Radiology, University of Washington, Seattle, USA

**Keywords:** Carotid artery, Atherosclerosis, Intraplaque hemorrhage, Acute cerebral infarct, Cardiovascular magnetic resonance imaging, Magnetic Resonance Imaging (MRI), Ischemic stroke, Atherosclerosis, Stenosis, Vascular disease

## Abstract

**Background:**

To determine the usefulness of the size of carotid artery intraplaque hemorrhage (IPH) in discriminating the risk of acute ischemic stroke using cardiovascular magnetic resonance (CMR) vessel wall imaging.

**Methods:**

Symptomatic patients with carotid atherosclerotic plaque who participated in a cross-sectional, multicenter study of CARE-II (NCT02017756) were included. All patients underwent carotid and brain CMR imaging. Carotid plaque burden and the size of plaque compositions including calcification, lipid-rich necrotic core (LRNC), and IPH were measured. Presence of acute cerebral infarct (ACI) in ipsilateral hemisphere of carotid plaque was determined. The relationship between carotid plaque features and presence of ipsilateral ACI was then analyzed.

**Results:**

Of 687 recruited patients (62.7 ± 10.1 years; 69.4% males) with carotid plaque, 28.5% had ACI in ipsilateral hemispheres. Logistic regression revealed that carotid plaque burden was significantly associated with the presence of ACI before and after adjusted for clinical confounding factors. The volume of LRNC, %LRNC volume, volume of IPH, and %IPH volume were significantly associated with ACI before (volume of LRNC: OR = 1.297, *p* = 0.005; %LRNC volume: OR = 1.119, *p* = 0.018; volume of IPH: OR = 2.514, *p* = 0.003; %IPH volume: OR = 2.202, *p* = 0.003) and after (volume of LRNC: OR = 1.312, *p* = 0.006; %LRNC volume: OR = 1.90, *p* = 0.034; volume of IPH: OR = 2.907, *p* = 0.007; % IPH volume: OR = 2.374, *p* = 0.004) adjusted for clinical confounding factors. The association between volume of IPH and ACI remained statistically significant after further adjusted for plaque volume (OR = 2.813, *p* = 0.016) or both plaque volume and volume of LRNC (OR = 4.044, *p* = 0.024).

**Conclusions:**

In symptomatic patients with carotid atherosclerotic plaques, the size of IPH is independently associated with ipsilateral ACI, suggesting the size of IPH might be a useful indicator for the risk of ACI.

**Trial registration:**

Clinical Trial Registration-URL: http://www.clinicaltrials.gov. Unique Identifier: NCT02017756.

## Background

Previous studies have shown that presence of intraplaque hemorrhage (IPH) in carotid arteries was a strong predictor for future ischemic events [1, 2]. The underlying mechanism might be based on the hypothesis that IPH accelerates the progression of atherosclerotic plaque and increases the risk of plaque rupture. Physiopathologically, the extracorpuscular hemoglobin released from the phagocytosis of red blood cells promotes local inflammation and proteolytic enzymes that accelerate the degradation and subsequent disruption of fibrous cap [3]. With the increases in the amount of IPH, the effect of inflammatory factors and proteolytic enzymes might be amplified hypothetically. A most recent study by Wang et al. reported that symptomatic carotid plaques tended to having marginally larger volume of IPH compared to those with asymptomatic carotid plaques [4]. However, the usefulness of the size of IPH for determining the risk of acute cerebral infarct (ACI) remains unclear.

Mutlicontrast cardiovascular magnetic resonance (CMR) vessel wall imaging has been demonstrated to be capable of accurately assessing the morphological and compositional characteristics of carotid atherosclerotic plaques [5]. Among three T1 weighted imaging sequences of time-of-flight (TOF), T1-weighted (T1W), and magnetization-prepared rapid acquisition gradient echo (MPRAGE), MPRAGE showed the best agreement with histology in evaluating carotid IPH [6]. Therefore, adding an MPRAGE sequence into the multicontrast vessel wall imaging protocol will improve the accuracy of the assessment of carotid IPH.

The aim of this study was to determine the usefulness of the size of carotid IPH for determination of risk of ACI in symptomatic patients with carotid plaques using multi-contrast CMR vessel wall imaging. Since all patients with IPH will be classified into the same risk of developing stroke according to previous evidences, this study would provide additional information for assessing the stroke risk using the size of IPH.

## Methods

### Study population

**Fig. 1:**
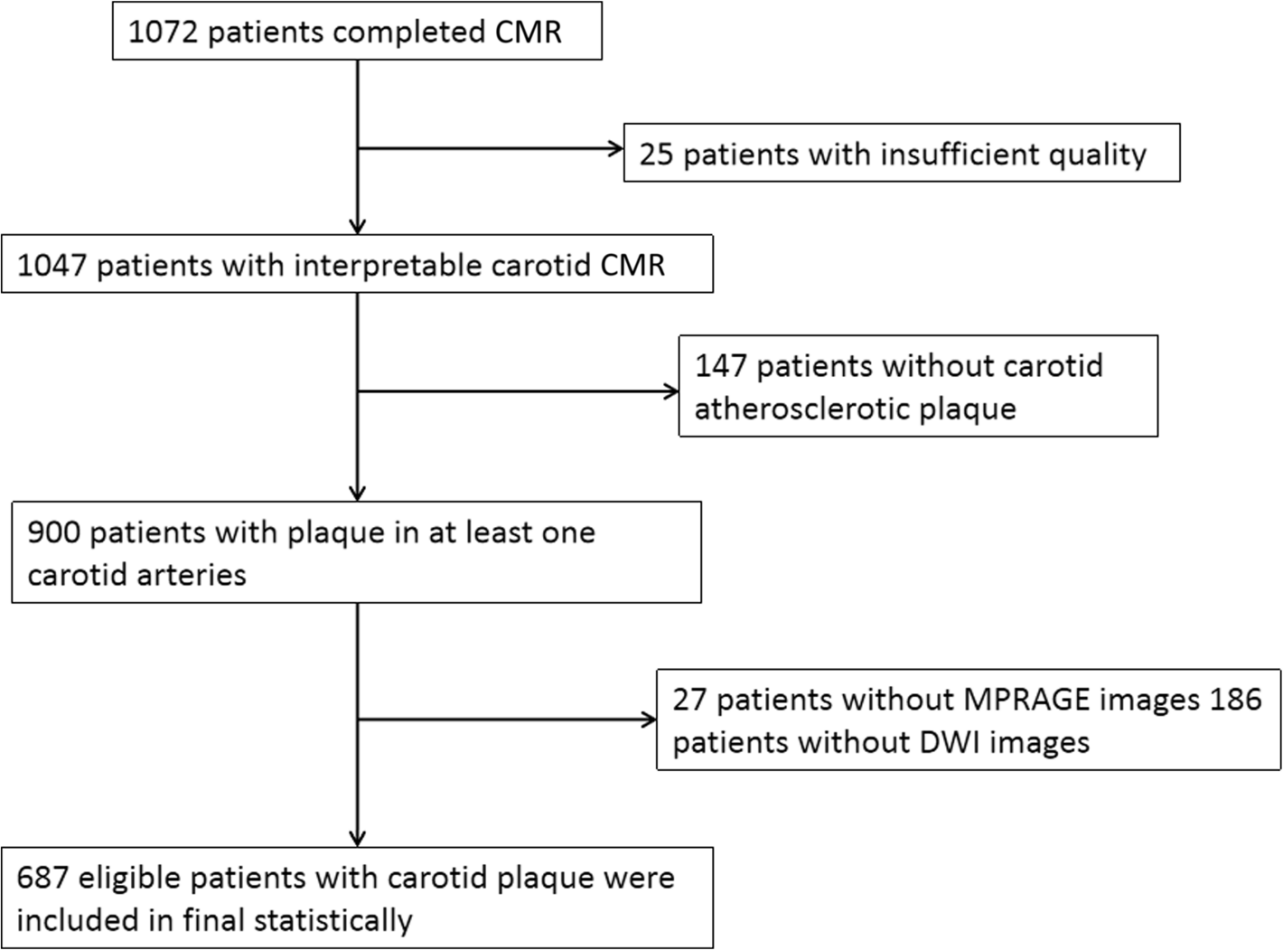
Flow chart of patient recruitment for final analysis

The patients were recruited from a cross-sectional, multicenter, observational study of Chinese Atherosclerosis Risk Evaluation (CARE-II, NCT02017756) which aimed to determine the prevalence of carotid artery high-risk atherosclerotic plaque in symptomatic patients in China. The study design had been published [7]. The inclusion criteria of CARE-II study were as follows: 1) 18 to 80 years; 2) had recent (< 2 weeks) cerebrovascular ischemic events (ischemic stroke or transient ischemic attack); and 3) had atherosclerotic plaques in at least one carotid artery identified by ultrasound imaging. Subjects with cardioembolic stroke, hemorrhage stroke, history of radiation therapy in the neck, or contraindication to CMR examination were excluded. A flow chart of patient recruitment is presented in Fig. 1. The following clinical data were collected from the medical records: age, gender, body mass index (BMI), blood pressure, lipid panel results including total cholesterol, low-density lipoprotein cholesterol (LDL), high-density lipoprotein cholesterol, and triglycerides, history of hypertension, smoking, hyperlipidemia, diabetes, and coronary artery disease (CAD). The study protocol was approved by local Ethics Committee at each participating institution and all patients provided written informed consent.

### CMR imaging protocol

CMR was performed on 3 T CMR scanners (Achieva TX, Philips Healthcare, Best, The Netherlands) with 8-channel phase array carotid coils and 8-channel head coils. All the imaging sites utilized CMR scanners with the same vendor and model. The carotid artery vessel wall was imaged by multicontrast imaging protocol including three dimensional (3D) TOF, two-dimensional T1W and T2-weighted (T2W), and 3D MPRAGE sequences with the following parameters: TOF: fast field echo (FFE), repeat time (TR) / echo time (TE) 20 ms/4.9 ms, flip angle 20°, field of view (FOV) 14 × 14 cm^2^, matrix size 256 × 256, and slice thickness 1 mm; T1W: turbo spin echo (TSE), TE/TR 800 ms/10 ms, FOV 14 × 14 cm^2^, matrix size 256 × 256, and slice thickness 2 mm; T2W: TSE, TE/TR 4800 ms/50 ms, FOV 14 × 14 cm^2^, matrix size 256 × 256, and slice thickness 2 mm; and MPRAGE: FFE, TE/TR 8.8 ms/5.3 ms, flip angle 15°, FOV 14 × 14 cm^2^, matrix size 256 × 256, and slice thickness 1 mm. A clinically routine imaging protocol was used to acquire brain images with the following parameters: T1W: FFE, TE/TR 308 ms/4.6 ms, FOV 23 × 23 cm^2^, matrix size 400 × 256, and slice thickness 5.5 mm; T2W: TSE, TE/TR 3000 ms/80 ms, FOV 23 × 23 cm^2^, matrix size 400 × 255, and slice thickness 5.5 mm; and diffusion weighted imaging (DWI): echo planar imaging, TR/TE 2724 ms/86 ms, FOV 23 × 23 cm^2^, matrix size 128 × 126, and slice thickness 5.5 mm. The gadolinium-based contrast agent was not administrated for carotid CMR imaging in this study.

### CMR image analysis

CMR images were reviewed by experienced radiologists (> 3 years’ experience in neuroradiology) with consensus who were blinded to clinical information. Brain MR images were reviewed by two radiologists (Y.L. and M.W.) who were blinded to carotid images. Presence or absence of ACI in bilateral hemispheres was determined respectively. The ACI was defined as lesion which shows hyperintensity on DWI images but iso- or hypointense on T1W images.

The carotid vessel wall images were interpreted by another two radiologists (Y.H. and X.Z.) who were blinded to brain images using a custom-designed software (CASCADE, University of Washington, Seattle, Washington, USA). The boundaries of lumen, outer wall and plaque components were outlined manually at each axial location of carotid arteries for measuring the morphology including lumen area, wall area, mean wall thickness (mean WT), IPH area, lipid-rich necrotic core (LRNC) area, and calcification area. Then total vessel area (total vessel area = lumen area + wall area), and normalized wall index (NWI = wall area / total vessel area × 100%) were calculated. For each subject, the morphological measurements were taken from the mean values of each corresponding measurement. The volume of plaque and each plaque component were calculated as the sum of plaque area and each plaque component area with corresponding slices multiplied by slice thickness respectively. The percentage of the volume of each plaque component divided by plaque volume was calculated. Carotid atherosclerotic plaque was defined as eccentric wall thickening on CMR vessel wall images. Presence or absence of atherosclerotic plaque components including calcification, LRNC, IPH, and fibrous cap rupture (FCR) in carotid arteries were identified using published criteria [8]. Calcification was characterized by the hypointense on TOF, T1W, T2W, and MPRAGE images. The LRNC showed isointense on TOF and T1W images and hypointense on T2W images within the plaque. The IPH will be determined when there was hyperintense on TOF, T1W, and MPRAGE (1.5 times of signal intensity compared to muscle) images. FCR was identified when there was deficit in fibrous cap or discontinuous surface of the plaque. For arteries with IPH, the age of IPH was also evaluated using the following criteria: fresh IPH: intact red blood cell with intracellular methemoglobin, which showed hyperintense on T1W or 3D TOF images and hypointense or isointense on T2W images; and recent IPH: lytic red blood cell with extracellular methemoglobin, which showed hyperintense on T1W or 3D TOF images and hyperintense on T2W images [9]. The luminal stenosis of carotid artery was measured on the reconstructed images of TOF CMR angiography with maximum intensity project algorism using North American Symptomatic Carotid Endarterectomy Trial (NASCET) criteria [10].

### Statistical analysis

Continuous variables were summarized as mean ± standard deviation (SD) and the categorical variables were described as percentage. For patients with bilateral carotid plaques, the CMR imaging features in plaques with larger plaque burden (plaque volume) were selected for analysis. The clinical characteristics and plaque features in carotid artery were compared between patients with and without ipsilateral ACI using independent-sample *t* test, Mann-Whitney U test, Chi-square test, or Fisher’s test as appropriate. The odds ratio (OR) and corresponding 95% confidence interval (CI) of plaque features in discriminating ACI were calculated using logistic regression before and after adjusted for confounding factors. The confounding factors include age, gender, and those clinical factors which were associated with presence of ACI. Two-sided tests were used and *p* value < 0.05 was considered as statistically significant. All statistical analyses were performed using SPSS 16.0 (Statistical Package for the Social Sciences (SSPS) International Business Machines, Inc., Armonk, New York, USA).

## Results

### Characteristics of the study population

Figure 1 represents the flow chart of the patient recruitment. Of 1047 patients of CARE-II study, 900 were found to have plaque in at least one carotid artery. Of the remaining 900 patients, 213 were excluded due to the following reasons: 1) no MPRAGE images or with poor MPRAGE images (*n* = 27); 2) no DWI images (*n* = 186). Of 687 eligible subjects (62.7 ± 10.1 years, 477 (69.4%) males, 516 (75.1%) had hypertension, 380 (55.3%) had hyperlipidemia, 216 (31.4%) had diabetes, 347 (50.5%) had smoke history, and 109 (15.9%) had CAD. Compared with patients without ACI, those with ACI had significantly higher BMI (24.9 ± 3.1 vs. 24.2 ± 3.1 kg/m^2^, *p* = 0.010), younger age (60.9 ± 9.8 vs. 63.3 ± 10.1 years, *p* = 0.005), higher prevalence of males (78.6 vs. 65.8%, *p* = 0.001), smoke (58.2 vs. 47.5%, *p* = 0.011), and diabetes (39.3 vs. 28.3%, p = 0.005), and lower LDL (2.8 ± 0.9 vs. 3.1 ± 1.0 years, *p* = 0.004). The demographic and clinical characteristics are detailed in Table 1.

**Table 1:** Comparison of clinical characteristics of study population

	Mean ± SD or n (%)		p
	Patients with ACI	Patients without ACI	
	(*n* = 196)	(*n* = 491)	
Age, years	60.9 ± 9.8	63.3 ± 10.1	0.005
Gender, male	154 (78.6)	323 (65.8)	0.001
BMI, kg/m^2^	24.9 ± 3.1	24.2 ± 3.1	0.010
Smoke	114 (58.2)	233 (47.5)	0.011
Diabetes	77 (39.3)	139 (28.3)	0.005
Hypertension	149 (76.0)	367 (74.7)	0.727
SBP, mmHg	145.8 ± 24.7	144.7 ± 20.7	0.999
DBP, mmHg	88.4 ± 13.7	86.9 ± 13.4	0.138
Hyperlipidemia	108 (55.1)	272 (55.4)	0.944
TC, mmol/L	4.5 ± 1.2	4.7 ± 1.1	0.093
TG, mmol/L	1.9 ± 1.2	1.8 ± 1.0	0.833
HDL, mmol/L	1.1 ± 0.4	1.1 ± 0.3	0.089
LDL, mmol/L	2.8 ± 0.9	3.1 ± 1.0	0.004
CAD	34 (17.3)	75 (15.3)	0.502

*ACI* Acute cerebral infarction, *BMI* body mass index, *SBP* systolic blood pressure, *DBP* diastolic blood pressure, *LDL* low density lipoprotein, *HDL* high density lipoprotein, *TC* total cholesterol, *TG* triglycerides, *CAD* coronary artery disease

### Comparison of plaque features between patients with and without ACI

The morphological and compositional characteristics of carotid plaque are detailed in Table 2. Of 687 patients with plaque in carotid arteries, 196 (28.5%) had ACI in the ipsilateral hemispheres. Compared with patients without ACI, those with ACI had significantly greater carotid wall area (35.4 ± 11.6 vs. 32.5 ± 11.0 mm^2^, *p* = 0.002), total vessel area (80.7 ± 19.5 vs. 77.0 ± 20.7 mm^2^, *p* = 0.016), mean WT (1.3 ± 0.5 vs. 1.2 ± 0.4 mm, *p* = 0.007), and marginally larger luminal stenosis (26.4 ± 40.0% vs. 16.1 ± 27.5%, *p* = 0.058). No significant differences were found in the lumen area, normalized wall index, and plaque volume between patients with and without ACI (all *p* > 0.05).

**Table 2:** Comparison of carotid plaque features between patients with and without ACI

	Mean ± SD, or n (%)		p
	Patients with ACI (*n* = 196)	Patients without ACI (*n* = 491)	
Plaque morphology			
Lumen area, mm^2^	45.3 ± 15.2	44.4 ± 14.9	0.443
Wall area, mm^2^	35.4 ± 11.6	32.5 ± 11.0	0.002
Total vessel area, mm^2^	80.7 ± 19.5	77.0 ± 20.7	0.016
Mean wall thickness, mm	1.3 ± 0.5	1.2 ± 0.4	0.007
Normalized wall index, %	44.2 ± 10.7	42.6 ± 9.3	0.122
Luminal stenosis, %	26.4 ± 40.0	16.1 ± 27.5	0.058
^a^ Plaque volume, mm^3^	722.5 ± 475.3	667.3 ± 426.9	0.320
Presence of plaque compositions			
Calcification	101 (51.5)	247 (50.3)	0.772
Fibrous cap rupture	18 (9.2)	40 (8.1)	0.659
LRNC	148 (75.5)	380 (77.4)	0.597
All IPH	42 (21.4)	76 (15.5)	0.062
Fresh IPH	22 (11.2)	38 (0.8)	0.804
Recent IPH	8 (0.4)	23 (0.5)	0.185
Both two types of IPH	12 (0.6)	15 (0.3)	0.274
^b^ Size of plaque compositions			
Volume of calcification, mm^3^	37.1 ± 76.2	38.7 ± 51.8	0.194
% calcification volume, %	3.3 ± 4.2	4.0 ± 4.0	0.046
Volume of LRNC, mm^3^	166.5 ± 243.9	113.4 ± 153.7	0.083
% LRNC volume, %	14.9 ± 14.2	12.1 ± 11.1	0.105
Volume of IPH, mm^3^	164.9 ± 200.5	71.4 ± 67.7	0.006
% IPH volume, %	12.1 ± 10.5	7.0 ± 5.9	0.014

*LRNC* lipid-rich necrotic core, *IPH* intraplaque hemorrhage. ^a^ The value was calculated from the arterial slices with atherosclerotic plaque. ^b^ The value was included from the arterial slices with the corresponding plaque composition, respectively

**Fig. 2:**
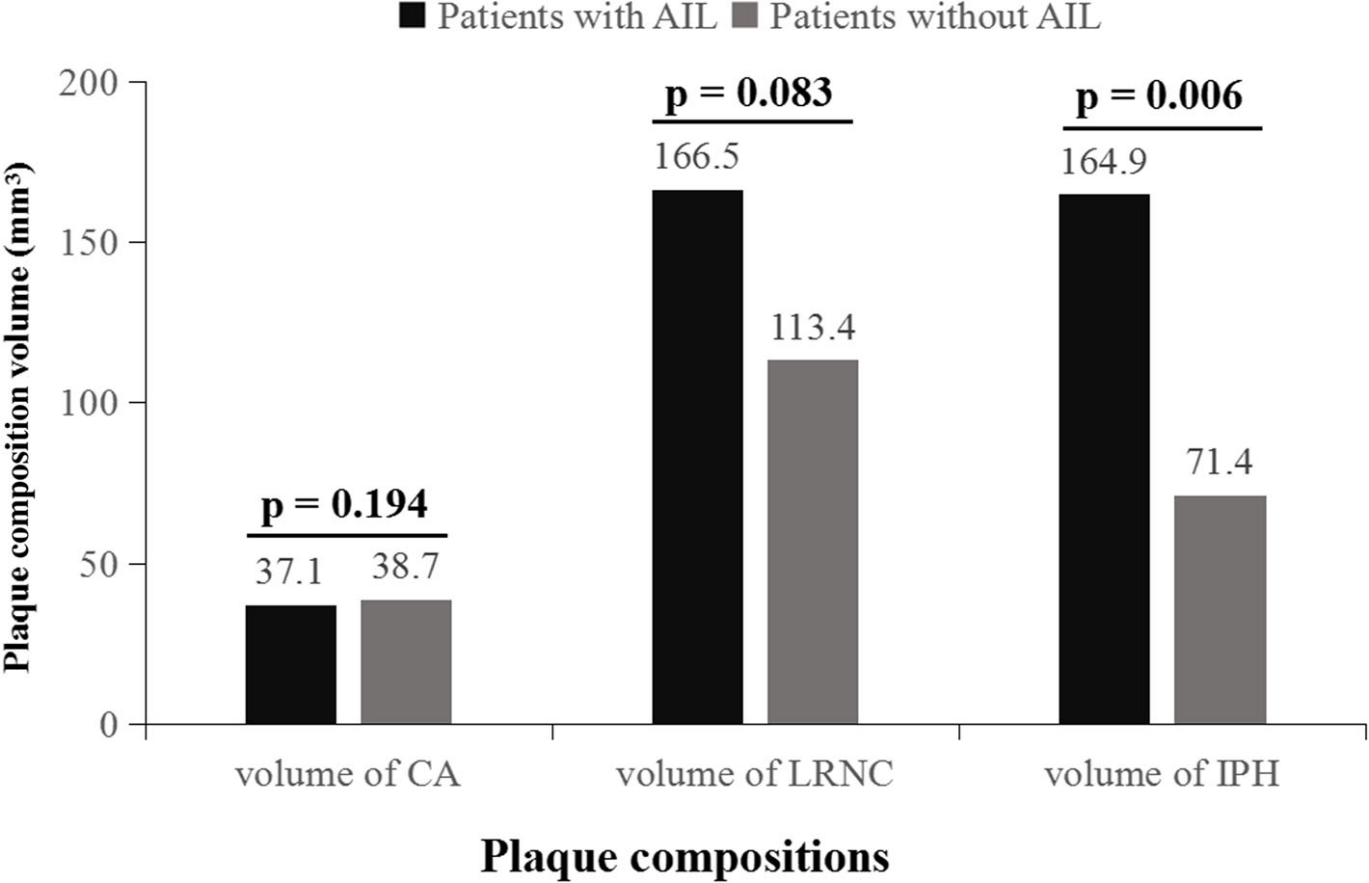
A bar graph of volume of carotid plaque components including calcification (CA), lipid-rich necrotic core (LRNC), and intraplaque hemorrhage (IPH). Volume of and IPH was significantly associated with the presence of acute cerebral infarct

In this study population, of all 687 subjects, 348 (50.7%) had calcification, 528 (76.9%) had LRNC, 118 (17.2%) had IPH, and 58 (8.4%) had FCR. Patients with ACI had marginally higher prevalence of IPH (21.4% vs. 15.5%, *p* = 0.062) compared with those without ACI. No significant differences in presence of calcification, LRNC, and FCR between subjects with and without ACI (all *p* > 0.05). Patients with ACI had significantly larger volume of IPH (164.9 ± 200.5 vs. 71.4 ± 67.7 mm^3^, *p* = 0.006) (Fig. 2), larger percent of IPH volume (12.1 ± 10.5% vs. 7.0 ± 5.9%, *p* = 0.014), and lower percent of calcification volume (3.3 ± 4.2% vs. 4.0 ± 4.0%, *p* = 0.046) compared with those without ACI. There were no significant differences in the volume of calcification (Fig. 2), volume of LRNC (Fig. 2), and percent of LRNC volume between patients with and without ACI (all *p* > 0.05). Of 118 subjects who had carotid plaques with IPH, 60 (50.8%) had fresh IPH, 31 (26.3%) had recent IPH, and 27 (22.9%) had both fresh and recent IPHs. There were no significant differences in the prevalence of fresh IPH, recent IPH and both two types of IPH between patients with and without ACI (all *p* > 0.05).

### Association of carotid plaque features with ipsilateral ACI

**Fig. 3:**
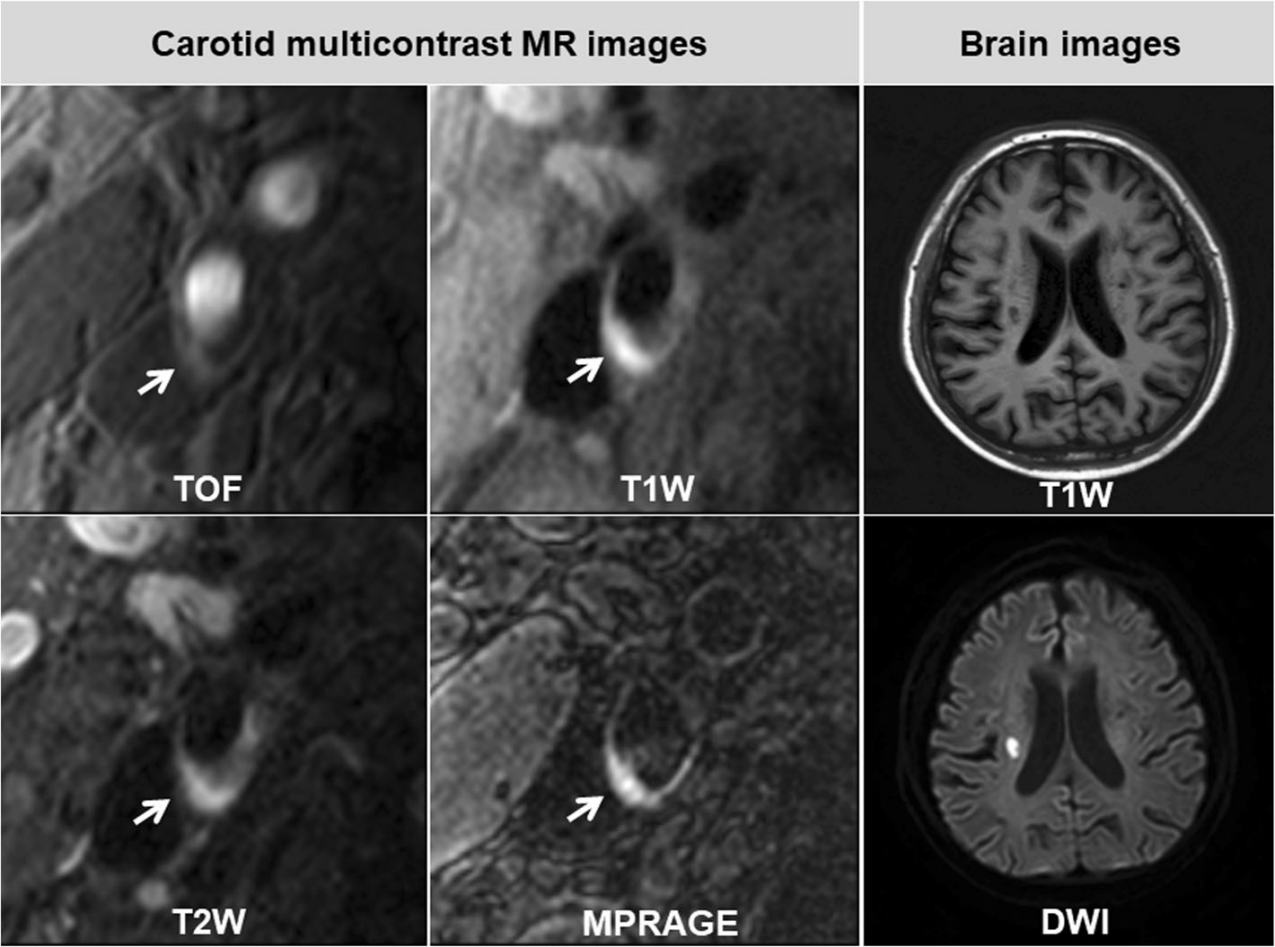
An example of a patient with carotid intraplaque hemorrhage (IPH) and ipsilateral acute cerebral infarct (ACI). Multicontrast MR images show an atherosclerotic plaque with large IPH (hyperintensity within the vessel wall, white arrows) in the right internal carotid artery. On Brain images of the same patient, acute cerebral infarct which shows hypointensity on T1W image and hyperintensity on DWI image can be seen in the right hemisphere

The results of logistic regression analysis are detailed in Table 3. Univariate logistic regression showed that wall area (OR, 1.269; 95%CI, 1.081–1.489; p = 0.014), total vessel area (OR, 1.194; 95%CI, 1.014–1.406; *p* = 0.034), mean WT (OR, 1.293; 95%CI, 1.103–1.515; *p* = 0.002), volume of LRNC (OR, 1.297; 95%CI, 1.084–1.553; *p* = 0.05), and volume of IPH (OR, 2.514; 95% CI, 1.363–4.637; *p* = 0.003) with increment of 1 SD, and luminal stenosis (OR, 1.098; 95%CI, 1.044–1.155; *p* < 0.001), % LRNC volume (OR, 1.199; 95%CI, 1.032–1.393; *p* = 0.018), and %IPH volume (OR, 2.202; 95%CI, 1.317–3.680; *p* = 0.003) with increment of 10% were significantly associated with presence of ACI. Multivariate logistic regression revealed that the associations of ACI with wall area, mean WT, luminal stenosis, volume of LRNC, % LRNC volume, volume of IPH, and % IPH volume remained statistically significant after adjusted for clinical confounding factors of age, gender, BMI, hypertension, hyperlipidemia, diabetes, and smoke, and CHD (model 1). After further adjusted for plaque volume (model 2), significant associations of presence of ACI with volume of LRNC (OR, 1.398; 95% CI, 1.045–1.870; *p* = 0.024) and volume of IPH (OR, 2.813; 95% CI, 1.212–6.528; *p* = 0.016) can be found (Table 3). Moreover, the association of ACI with volume of IPH (OR, 4.044; 95% CI, 1.200–13.626; p = 0.024) remained statistically significant after adjusted for the clinical risk factors, plaque volume, and volume of LRNC. In contrast, the association of ACI with LRNC volume was not statistically significant after adjusted for clinical risk factors, plaque volume, and volume of IPH. There were no significant associations between the presence of calcification, FCR, LRNC, and IPH, volume of calcification, and percent volume of calcification and the presence of ACI before and after adjusting for confounding factors (all *p* > 0.05). Figure 3 represents an example of a patient who had large volume of IPH in carotid artery and ipsilateral ACI. In addition, logistic regression showed that the presence of carotid IPH (OR, 15.698; 95% CI, 8.575–28.739; *p* < 0.001) and volume of IPH with increment of 1 SD (OR, 3.322; 95% CI, 1.685–6.548; *p* = 0.001) were significantly associated with presence of FCR.

**Table 3:** Association of carotid plaque features with ipsilateral ACI

	Presence of ACI								
	Univariate regression			Multivariate regression model 1^d^			Multivariate regression model 2 ^e^		
	OR	95% CI	p	OR	95% CI	p	OR	95% CI	p
^a^ Lumen area, mm^2^	1.058	0.898–1.247	0.501	0.975	0.819–1.161	0.776			
^a^ Wall area, mm^2^	1.269	1.081–1.489	0.004	1.257	1.047–1.510	0.014			
^a^ Total vessel area, mm^2^	1.194	1.014–1.406	0.034	1.109	0.924–1.331	0.268			
^a^ Mean wall thickness, mm^2^	1.293	1.103–1.515	0.002	1.315	1.103–1.568	0.002			
^b^ NWI, %	1.177	0.997–1.390	0.054	1.202	1.008–1.434	0.040			
^a^ Plaque volume, mm^3^	1.130	0.961–1.329	0.140	1.087	0.903–1.308	0.378			
^b^ Luminal stenosis, %	1.098	1.044–1.155	< 0.001	1.096	1.039–1.155	0.001			
Presence of calcification	1.050	0.754–1.463	0.772	1.094	0.757–1.582	0.632	1.024	0.680–1.541	0.910
Presence of FCR	1.140	0.637–2.042	0.659	1.183	0.646–2.167	0.586	1.095	0.578–2.074	0.780
Presence of LRNC	0.901	0.611–6.328	0.597	0.780	0.516–1.180	0.240	0.663	0.417–1.054	0.083
Presence of IPH	1.489	0.979–2.266	0.063	1.515	0.963–2.384	0.072	1.485	0.907–2.432	0.116
^c^ Volume of calcification, mm^3^	0.971	0.763–1.234	0.808	1.023	0.794–1.318	0.859	0.953	0.702–1.294	0.759
^c^ % calcification volume, %	0.605	0.314–1.166	0.133	0.731	0.372–1.436	0.363			
^c^ Volume of LRNC, mm^3^	1.297	1.084–1.553	0.005	1.312	1.082–1.591	0.006	1.398	1.045–1.870	0.024
^c^ % LRNC volume, %	1.199	1.032–1.393	0.018	1.190	1.014–1.398	0.034			
^c^ Volume of IPH, mm^3^	2.514	1.363–4.637	0.003	2.907	1.341–6.301	0.007	2.813	1.212–6.528	0.016
^c^ % IPH volume, %	2.202	1.317–3.680	0.003	2.374	1.324–4.257	0.004			

*NWI* normalized wall index, *FCR* fibrous cap rupture, *LRNC* lipid-rich necrotic core, *IPH* intraplaque hemorrhage. ^a^ The increment for plaque burden was 1 SD. ^b^ The increment for NWI and luminal stenosis was 10% NWI and stenosis. ^c^ Only for patients with the corresponding plaque compositional feature with increment of 1 SD for composition size and 10% for percent of composition volume. ^d^ Model 1: adjustment for age, gender, BMI, hypertension, hyperlipidemia, CHD, diabetes, and smoke. ^e^ Model 2: adjustment for the former clinical factors and plaque volume

## Discussion

The present study investigated the relationship between atherosclerotic plaque characteristics and risk of ACI in symptomatic patients with carotid plaque using multicontrast CMR vessel wall imaging. We found that both carotid plaque burden and the size of IPH and LRNC were significantly associated with the presence of ipsilateral ACI before and after adjusted for age, gender, BMI, smoke, diabetes, hyperintense, hyperlipidemia, and CAD. In addition, after further adjusted for plaque volume and volume of LRNC, the association between volume of IPH and presence of ACI remained statistically significant. Our findings suggest that the association between volume of IPH and risk of ACI was independent of plaque burden and LRNC volume.

In this study, plaque burden in carotid artery with atherosclerotic plaque was found to be associated with the risk of ACI. The relationship between carotid plaque burden and stroke risk has been well demonstrated in previous studies. The degree of carotid stenosis is recognized as an important risk factor for cerebral infarction. Gunduz et al. studied 52 patients with 50–69 and > 70% carotid stenosis and found that the occurrence of new ischemic lesions was significantly related to carotid stenosis determined by carotid ultrasonography [11]. Beyond luminal stenosis, investigators also found that the vessel wall burden measured by ultrasound and CMR was associated with stroke risk. In a study by Liu et al., carotid plaque burden including mean WT (1.0 ± 0.2vs. 1.1 ± 0.4 mm, *p* = 0.043), mean wall area (26.2 ± 5.6 vs. 29.8 ± 9.8 mm^2^, *p* = 0.043), and percent volume of wall volume (35.5 ± 7.4 vs. 40.7 ± 11.9%, *p* = 0.021) was found to be greater in patients with recurrent stroke than that in those with first-time stroke [12]. Zhao et al. reported that carotid morphological measurements, such as maximum WT (β = 11.1;95% CI, 3.4–19.2; *p* = 0.005) and % wall volume (β = 33.5;95% CI, 14.1–56.2; *p* = 0.001), were significantly associated with ipsilateral acute cerebral infarction volume [13]. Our findings further compel the evidence that carotid plaque burden is an independent indicator for risk of ACI.

In the present study, volume of LRNC and % LRNC volume can be observed to be correlated with the presence of ACI before and after adjusted for clinical factors. Moreover, the association between presence of ACI and volume of LRNC was remained statistically significant after further adjusted for plaque volume. However, this association was not statistically significant after adjusted for clinical risk factors, plaque volume, and volume of IPH. This may be due to that enlargement of IPH volume would augment the size of LRNC. IPH was assumed to occur within LRNC. Hence, the size of LRNC was heavily driven by the size of IPH in our data. Investigators found that IPH can accelerate the progression of carotid plaques and LRNC size may govern the risk of future surface disruption, which was associated with a higher overall volume of cerebral injury [14–16]. Zhao et al. reported that LRNC volume was significantly associated with severity of cerebral infarction as measured by DWI lesions [13]. In addition, in the present study, we found that the presence of LRNC was insignificantly related to ACI, which is consistent with previous studies [17]. Our findings suggest that the size of LRNC is an effective indicator for the risk of ACI independent of plaque size but not IPH volume.

We found that the association of IPH volume in carotid arteries with ACI was independent of plaque size and LRNC volume. Moreover, logistic regression revealed that size of IPH had higher OR value than the size of LRNC, suggesting that IPH was more dangerous than fat loading of the plaque in determining the risk of ACI. This suggests that in carotid plaques with similar volume, the size of IPH may be used to further determine the risk of ACI. We also found that the presence of IPH was marginally significantly associated with ACI. A study using integrated backscatter ultrasound and CMR demonstrated that carotid plaques in the early stage after the onset of symptoms had a higher percent volume of lipid pool and IPH (57.8 ± 25.1% vs. 46.8 ± 25.1%, *p* = 0.036) compared with plaques that were stabilized by treatment for 30–180 days after the onset of symptoms [18]. In a most recent study, investigators found the symptomatic carotid plaques tended to having larger volume of IPH compared with asymptomatic plaques (150 ± 199 vs. 88 ± 106 mm^3^, *p* = 0.071) but the difference was not statistically significant which may be due to the smaller sample size (*n* = 31) [4]. As mentioned above, the presence of IPH might be a stimulator for plaque progression and future fibrous cap rupture [14, 19, 20]. A study by Cui et al. documented that volume of IPH was associated with minor fibrous cap disruption in carotid arteries (OR, 2.867; 95% CI, 1.505–5.461; *p* = 0.001) after adjusted for clinical confounding factors and plaque burden [21]. The inflammatory activity, coagulation proteases, leukocyte serine proteases, and gelatinase within atherosclerotic plaque with IPH may play important roles in degradation of fibrous cap and subsequent plaque rupture [3]. In addition, the biomechanical propriety of IPH might be contributable to the vulnerability of plaque surface. And a study by Sadat et al. found that hemorrhagic plaques had higher biomechanical stresses than non-hemorrhagic plaques [22]. To the best of our knowledge, disruption of carotid plaques triggers the formation of thromboembolus and subsequent ischemic stroke. In addition, some previous studies documented that no association can be found between the presence of IPH and ischemic stroke [23]. Our findings suggest that quantification of the size of IPH is warranted in patients with carotid plaques in stratification of stroke risk beyond the mere presence of IPH.

Previous studies showed that LDL participated in the promotion, development, and progression of atherosclerosis and LDL has been demonstrated to be an independent risk factor for ACI [24, 25]. However, our study found that patients with ACI had significantly lower LDL compared with patients without ACI. It may be due to the use of statins. A systematic and meta-analysis documented that statins can lower LDL cholesterol concentration [26].

There are several limitations in our study. First, this is a cross-sectional study that cannot determine the relationship between the progression of IPH volume in carotid arteries and ACI. Second, the current in-plane spatial resolution utilized in the present study was 0.55 mm which is limited to accurate quantify IPH with small size. To improve the spatial resolution is needed in future studies. Third, carotid vessel wall imaging of our study was performed with 2D multicontrast CMR imaging protocol with limited longitudinal coverage which may not capture the atherosclerotic plaque in more proximal or distal segments of extracranial carotid arteries. Wang et al. [27] proposed a simultaneous noncontrast angiography and intraplaque hemorrhage sequence with large longitudinal coverage [23] which could be used to evaluate carotid IPH in future studies.

## Conclusions

In patients with carotid atherosclerotic plaques, the size of IPH is independently associated with ipsilateral ACI, suggesting the size of IPH might be a useful indicator for the risk of ACI.

## Data Availability

All data generated or analysis during this study are included in this published article.

## Abbreviations

3D: Three dimensional
ACI: Acute cerebral infarct
BMI: Body mass index
CAD: Coronary artery disease
CI: Confidence interval
CMR: Cardiovascular magnetic resonance
DWI: Diffusion weighted imaging
FCR: Fibrous cap rupture
FFE: Fast field echo
FOV: Field of view
IPH: Intraplaque hemorrhage
LDL: Low density lipoprotein
LRNC: Lipid-rich necrotic core
MPRAGE: Magnetization-prepared rapid acquisition gradient echo
NASCET: North American Symptomatic Carotid Endarterectomy Trial
NWI: Normalized wall index
OR: Odds ratio
SD: Standard deviation
T1W: T1 weighted
T2W: T2 weighted
TE: Echo time
TOF: Time-of-flight
TR: Repetition time
TSE: Turbo spin echo
WT: Wall thickness
